# Retrospective

**Published:** 1882-09

**Authors:** 


### RETROSPECTIVE.

One year ago we assumed editorial management of the ATLANTA MEDICAL REGISTER, and in the initial number of the new series was recorded a pledge to the effect that this journal should be conducted upon an absolusely independent basis—that it was not, and while under our control, should never become, the organ or the instrument of any faction, firm, organization or institution; but that it should be so managed as to subserve, according to the dictates of our judgment, the best interests of the whole medical profession, with the hope, as then expressed, that it would be the means of conveying information, disseminating knowledge, promoting the truth, upholding the right, and cementing the ties of professional brotherhood.

With the present number the first annual volume is closed and we consider it appropriate to cast a retrospective glance over the labors of a twelvemonth, and review briefly the results of an honest and earnest endeavor to contribute to the pleasure and improvement of our readers.

It has been our constant aim to present, in each recurrent number of the journal, such a collection of practical matter, both original and selected, as would find a ready application at the hands of our friends in their every-day rounds of professional duties; and, in the editorial department, to give our views, for the most part, upon current topics of general interest. How well we have succeeded in the undertaking we must leave our readers to decide.

In reviewing new books, which have been sent to us for impartial notice, by authors and publishers, it has been our desire to deal out exact and equal justice to all, and so to present our criticisms that our readers would receive not only the opinions of an individual, but have before them the basis and reasons for that opinion, and thereby be enabled to judge for themselves as to the merits or defects of every work. We have carefully sought to make the review department a useful and an interesting feature of the REGISTER.

As to the variety of matter that has gone forth in this volume, we can do no better than refer to the general index which is hereto appended. It presents an alphabetibal synopsis of the contents for the year.

The present affords a fitting occasion to express to our friends our sincere appreciation of the kind manner in which our work has been received, and to return our sincere thanks for the cordial and substantial encouragement which they have so generously contributed.

We shall strive to become more and more worthy of their valued partiality, and with each successive volume to urge additional claims upon their friendly regard. But this is trending somewhat upon the Prospective, about which we shall have something to say in our next issue.

